# Case Report: Refractory Autoimmune Gastritis Responsive to Abatacept in LRBA Deficiency

**DOI:** 10.3389/fimmu.2021.619246

**Published:** 2021-02-26

**Authors:** Valentina Boz, Erica Valencic, Martina Girardelli, Alessia Pin, Laura Gàmez-Diaz, Alberto Tommasini, Sara Lega, Matteo Bramuzzo

**Affiliations:** ^1^Department of Medical, Surgical and Health Sciences, University of Trieste, Trieste, Italy; ^2^Department of Pediatrics, Institute for Maternal and Child Health-IRCCS “Burlo Garofolo”, Trieste, Italy; ^3^Center for Chronic Immunodeficiency, University Medical Center Freiburg, Freiburg im Breisgau, Germany

**Keywords:** autoimmune gastritis, lymphoproliferation, inflammatory bowel disease, LRBA deficiency, primary immumunodeficiencies, case report, abatacept

## Abstract

Primary immunodeficiency (PID) with immune dysregulation may present with early onset gastrointestinal autoimmune disorders. When gastrointestinal autoimmunity is associated with multiple extraintestinal immune system dysfunction the diagnosis of PID is straightforward. However, with the advent of next generation sequencing technologies, genetic defects in PID genes have been increasingly recognized even when a single or no extraintestinal signs of immune dysregulation are present. A genetic diagnosis is especially important considering the expanding armamentarium of therapies designed to inhibit specific molecular pathways. We describe a boy with early-onset severe, refractory autoimmune gastritis and biallelic mutations in the *LRBA* gene causing a premature STOP-codon who was successfully treated with CTLA4-Ig, abatacept, with long term clinical and endoscopic remission. The case underscores the importance to consider a monogenetic defect in early onset autoimmune disorders, since the availability of targeted treatments may significantly improve patient prognosis.

## Introduction

LPS responsive beige–like anchor protein (LRBA) deficiency is a primary immunodeficiency disease associated with immune dysregulation (1, 2). LRBA deficiency is characterized by a wide range of manifestations, including humoral immune deficiency, lymphoproliferation, hematologic and organ autoimmunity (3), and shares clinical features with inflammatory bowel disease, common variable immunodeficiency, and autoimmune lymphoproliferative syndrome. LRBA is a cytoplasmic protein involved in the regulation of intracellular vesicle trafficking and exocytosis (4). Biallelic mutations in *LRBA* lead to defective protein expression, resulting in altered recycling of cytotoxic T-lymphocyte associated protein 4 (CTLA4) on the membrane of regulatory T cells (5) hence the similarity with CTLA4 deficiency phenotype (6). Of note, both LRBA deficiency and CTLA4 deficiency can benefit from a biologic treatment with CTLA4-Ig (abatacept), which surrogates at least in part the defective CTLA4 function (7, 8). Hematopoietic stem cell transplantation can cure the disease and is a valuable option especially if performed before the development of severe organ damage (3, 8).

A prompt diagnosis of LRBA/CTLA-4 related disorders is thus of particular importance since the availability of targeted treatments can significantly improve patient prognosis.

## Case Report

We describe a boy with early onset autoimmune gastritis who was referred to our hospital for refractory disease. Since the age of 2 years the child had recurrent episodes of acute gastritis with hematemesis which at first were interpreted as the result of a gastric ulcer. At the age of four, due to persistence of recurrent vomiting with loss of weight and anemia he underwent a clinical work-up which showed a severe hemorrhagic gastritis with dense lymphocytic infiltrates in the lamina propria of the stomach and the duodenum. Laboratory investigation, including differential blood count and immunoglobulins, were normal. Celiac disease and Helicobacter pylori infection were ruled out. Inflammatory involvement of colon and terminal ileum was excluded.

The child received proton pump inhibitors (PPI), which were ineffective. Glucocorticoids therapy was started with clinical improvement, but steroid dependency was soon observed and the association of immunomodulating agents (first azathioprine, followed by methotrexate) was not effective in reducing the need for glucocorticoids.

At the time of our first visit, the boy was 7 years old. He had suspended medical treatments for a few months and relapsed. Gastric biopsies showed features of autoimmune gastritis with lymphocytic inflammation and apoptotic bodies, Anti-gastric wall antibodies were positive. Serum anti-transglutaminase IgA and IgG and anti-endomisium antibodies were repeatedly negative. Glucocorticoids were resumed in association with tacrolimus, and PPI, with partial clinical response.

In the following 2 years he remained dependent on high dose glucocorticoids and tacrolimus. Moreover, he developed recurrent episodes of unexplained fever, lasting 3–4 days, associated with painful cervical lymphadenopathy, and progressive splenomegaly. The finding of increased CD4- CD8- T lymphocytes with αβ T cell receptor (double negative T cells, DNT) supported a possible diagnosis of autoimmune lymphoproliferative syndrome (ALPS), even if no mutation was detected in the FAS gene. A therapeutic attempt with sirolimus to control systemic symptoms was soon stopped because of the occurrence of aphthous stomatitis. A slight reduction in fever recurrence was obtained after tonsillectomy.

**Figure 1:**
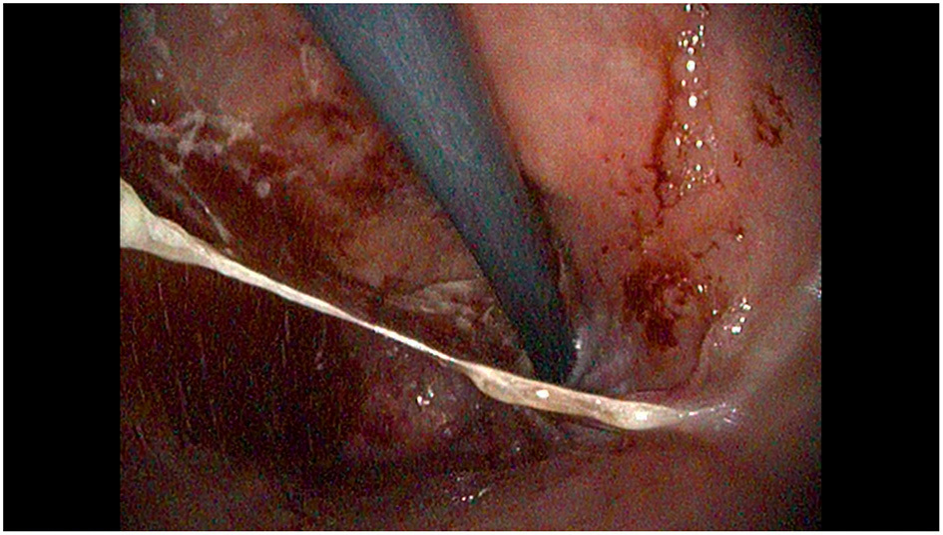
Retroverted view of the proximal stomach, showing diffuse mucosal congestion and hyperemia with discrete amounts of mucus and bleeding.

**Figure 2:**
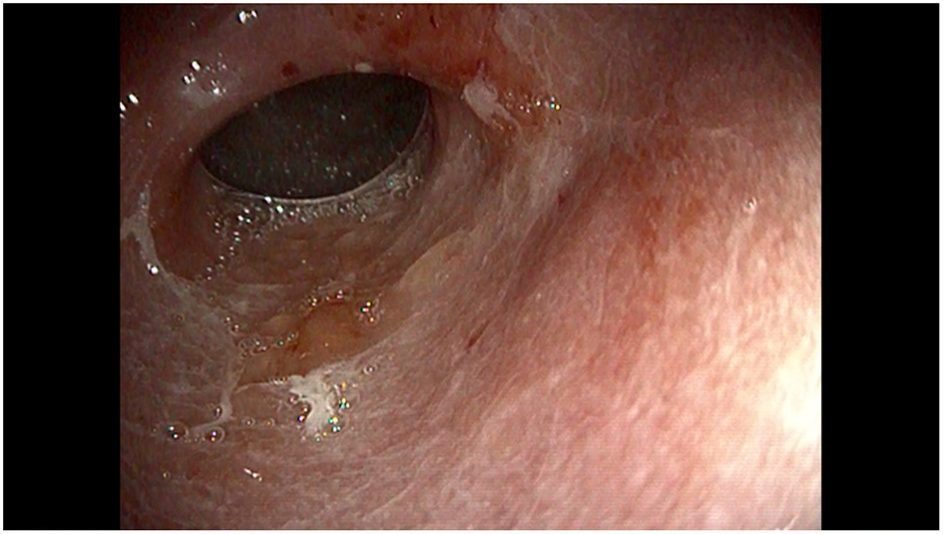
Endoscopic view of the distal stomach showing diffuse mucosal congestion, hyperemia and erosions.

At the age of 10 years, the child was evaluated because of the persistence of nausea, vomiting and hematemesis, despite treatment with tacrolimus. Blood counts showed microcytic anemia (Hb 8.7 g/dL, MCV 68 fL) with low ferritin (4.3 μg/L) and low vitamin B12 levels (178 pg/mL). Endoscopy showed diffuse mucosal hyperemia and congestion associated with erosions, ulcers, spots of bleeding and mucus deposits in the fundus, body and antrum suggestive for an active erosive gastritis (Figures 1, 2). No erosive lesions were found in the duodenum. Gastric biopsies showed markedly hyperplastic glandular elements within an inflamed lamina propria, with a prevalence of plasma cells and CD3 and CD8 positive T lymphocytes, infiltrating glands, hyperplasia of the surface epithelium and apoptotic bodies. T-lymphocyte infiltrates and plasma cells were also found in the lamina propria of the duodenum. The small and large bowel had no signs of inflammation.

Analysis of a gene panel for targeted sequencing of genes involved in immune dysregulation immunodeficiency, revealed biallelic mutations in the *LRBA* gene (chr 4; NM_006726; NP_006717). Both mutations (c.C6415T p.R2139X in exon 42 and c.C7315T p.R2439X in exon 49) cause a premature STOP-codon and can be classified as pathogenic (9). Variants are located in the PH and BEACH domain, respectively, and literature data described the deleterious effect of other non-sense mutations localized in these two domains (10). Cytometric evaluation showed a slight reduction of CTLA4 expression on peripheral lymphocytes, suggesting that the two mutations were associated with residual expression of LRBA (Supplementary Material). Gene expression analysis by RNA sequencing showed a down-regulation of LRBA in our patient compared to a group of healthy controls (Supplementary Material). Flow cytometry analysis showed that LRBA was expressed in cells from the patient, although at slightly lower levels than in controls. Since the mutation lies downstream of the domain recognized by the anti-LRBA polyclonal antibody we argued that the residual staining is due to the expression of a functionally defective protein, accounting for a reduced expression of CTLA4 on the cell membrane (Supplementary Material).

Based on this finding, treatment with subcutaneous abatacept (125 mg/weekly for the first 4 weeks, and then every 10 days) was started.

Within a few weeks, the boy had no more nausea or vomiting. After 6 months of treatment, anemia but not vitamin B12 deficiency resolved (Hb 12.4 g/dL, MCV 81.6 fL, ferritin 19.7 μg/L, vitamin B12 204 pg/mL) (Table 1).

**Table 1:** Laboratory data.

**Age**	**7**	**9**	**10**	**11**	**12**
Hemoglobin (g/dL)	13	13,5	8.7	12,4	13,6
MCV (fL)	74	82,9	68	81,6	85,8
Lymphocytes (cells/lL)	1,100	1,810	1,290	1,030	1,340
IgG (mg/dL)	560	735	649	547	1,103
IgA (mg/dL)	19	28	25	22	27
IgM (mg/dL)	118	103	104	103	186
Vitamin B12 (pg/mL)	231	174	178	204	262
Ferritin (mg/L)	nd	nd	4,3	19,7	17,6
T lymphocytes (%CD45++)	nd	79,1	76,7	87,2	82,8
T helper (%CD45++)	nd	52,1	48,5	64,1	57,7
T cytotoxic (%CD45++)	nd	19,1	20,5	17,1	19,7
RTE (%CD4+)	nd	26,3	38,3	35,1	54,6
DNT αβ(%CD3+)	nd	4,89	3,57	3,3	1,1
NK (%CD45++)	nd	8,9	13,4	5,8	9
B lymphocytes (%CD45++)	nd	8,2	6,4	4,1	6
IgM memory B lymphocytes (%CD19+)	nd	11,3	10,8	nd	4,5
Switched memory B lymphocytes (%CD19+)	nd	19,1	4,8	nd	2,5
Naïve B lymphocytes (%CD19+)	nd	56,8	77,7	nd	87,1

*DNT, Double Negative T cells; NK, Natural Killer; RTE, Recent Thymic Emigrants; nd, not done*.

**Figure 3:**
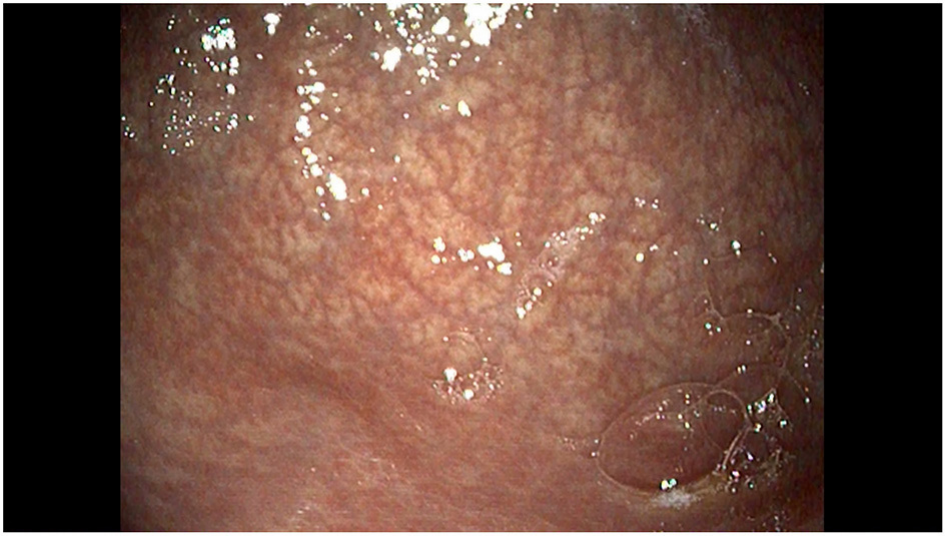
Endoscopic view of the large curvature of the stomach showing a normal mucosa with decreased gastric folds and increased visibility of the mucosal vascular pattern.

Endoscopy showed a gastric mucosa without erosive lesions, decreased gastric folds and an increased visibility of the vascular pattern in the antrum and in distal body of the stomach, consistent with atrophic gastritis (Figure 3). Histological evaluation showed mild activity of chronic gastritis with no apoptotic bodies in the glandular structures. Abatacept was continued while PPI was stopped.

Clinical, biochemical, macroscopic and microscopic findings remained unchanged after 12 and 18 months of treatment (Table 1).

## Discussion

LRBA deficiency is a protean disorder that can often present early in life with gastrointestinal complaints, in addition to infections, lymphoproliferation and organ or hematological autoimmunity (1, 10–16). It can represent the underpinning immune defect in cases of inflammatory bowel disease or autoimmune enteropathy with very-early onset (11, 17, 18). Histological features may include colonic crypt epithelium injury, chronic inflammation with remarkable increase of lymphocytes in the lamina propria and lymphocytic duodenitis resembling celiac disease (11, 19, 20). In our case, the main gastrointestinal complaint was a refractory erosive gastritis, without significant involvement of the remainder of the bowel apart from a lymphocytic duodenitis with villous atrophy, reminiscent of celiac disease. Indeed, LRBA deficiency has been associated with refractory celiac disease (21), but in our case celiac-related antibodies tested negative and a gluten free diet was not proposed. The gastric histology, together with the finding of autoantibodies, supported a diagnosis of autoimmune gastritis, which has not been described as a presenting feature in LRBA deficiency so far. Gastrointestinal manifestations have been described in case series of patients with LRBA deficiencies and include chronic diarrhea, chronic gastritis and inflammatory colitis resembling IBD (22). Also, case reports have shown LRBA deficiency in patients with IBD accompanied or not by antibody deficiency (19) and extraintestinal autoimmunity (11). Beside gastrointestinal autoimmunity, other manifestations of LRBA deficiency include hemolytic anemia, idiopathic thrombocytopenic purpura as well as type I diabetes and autoimmune hepatitis (12).

Differential diagnoses of LRBA deficiency include a variety of genetic defects with abnormalities of regulatory T cell function or production. Such defects are collectively identified as “IPEX-like disorders,” due to the coexistence of autoimmune enteropathy and immune dysregulation as in subjects with *FOXP3* mutations, and include CTLA4 haploinsufficiency, mutations in *STAT5b, STAT1, CD25*, and *ITCH* (23). CTLA-4 haploinsufficiency has significant clinical overlap with LRBA deficiency except for the fact that LRBA deficiency usually have lower levels of CTLA-4 expression and often manifests earlier. Patients with CTLA-4 haploinsufficiency may develop ALPS-like gastric and colonic involvement with predominance of lymphocytes and plasma cells infiltrates in the lamina propria (24). Patients with *STAT5b* mutations present with growth failure and pulmonary disease while *STAT1* mutations typically predispose to mucocutaneous candidiasis. Dysmorphic feature, developmental and growth delay and chronic lung disease are typical in *ITCH* mutation (23).

Autoimmune gastritis can also develop in adults with Common Variable Immunodeficiency (25), but our patient did not present humoral immunodeficiency at disease onset, as described in other cases (26). Of note, atrophic gastritis is associated with increased risk of gastric cancer both in the general population and in subjects with primary immunodeficiencies, and multifocal gastric adenocarcinoma has been reported in LRBA deficiency (27). In Italian adult subjects with Common Variable Immunodeficiency, gastric cancer is the leading cause of death (28). Thus, the availability of effective treatments to cure gastritis in primary immunodeficiencies is of crucial importance. In our patient, the only drugs allowing some relief from symptoms were glucocorticoids and tacrolimus, but a fairly good control of the disease was obtained only after treatment with abatacept, started on the basis of the genetic diagnosis. This is the first report showing that autoimmune gastritis associated with an immunodeficiency with immune dysregulation may respond to abatacept.

Our report carries a warning about the possibility that patients with isolated autoimmune or inflammatory signs, without clear immune disturbances, do not undergo a prompt and complete investigation in the suspicion of an immunodeficiency. In our case, genetic investigations were delayed due to normal routine laboratory investigations. However, an autoimmune lymphoproliferative syndrome (ALPS) was considered due to the development of lymphoproliferative symptoms with increased DNT, even if these features may be common to several primary immunodeficiencies, and have been described also di LRBA deficiency (29–31). Only later, the analysis of a panel of genes associated with immune dysregulation allowed to make the correct diagnosis. With the current wider availability of gene panels to diagnose rare monogenic disorders, and considering the wide genetic heterogeneity of immunodysregulation disorders, we should recommend genetic analysis in all children with early onset of autoimmune disorders, in the presence of red flags like atypical presentation for age or refractoriness to treatments, even in the absence of typical syndrome association with other immune disturbances.

In our case, the treatment with abatacept led to an almost complete control of symptoms. However, therapeutic choices in LRBA deficiency are controversial. Hematopoietic stem cell transplantation (HSCT) has been reported as the most effective treatment, mostly before patients show long-term severe clinical picture which may affect the success of the HSCT (3). In a large survey on LRBA deficiency, 24 patients out of 76 had been treated with HSCT at a mean age of 10 years, leading to complete remission or good response in 12/24, partial remission in five and death in seven. In the same survey, 52 patients had been treated with conventional therapy, including immunomodulators like sirolimus and abatacept, in most cases with partial good response. The probability of survival was similar in both treatment groups and was lower in subjects with lung involvement. Notably, the residual expression of LRBA protein was associated with 100% survival rate and with less severe disease manifestations (3). Indeed, complete absence of LRBA is associated with lower expression of CTLA4, as the protein is not recycled at all to the membrane (7). Thus, HSCT should be considered for patients with absent expression of LRBA and/or for subjects with severe or refractory disease manifestations.

Conversely, patients with residual expression of LRBA, or with near-normal expression of CTLA4, as in our case, can be selected for conservative treatments, based on immunomodulators and antimicrobial prophylaxis with antibiotics and immunoglobulins, if needed.

**Figure 4:**
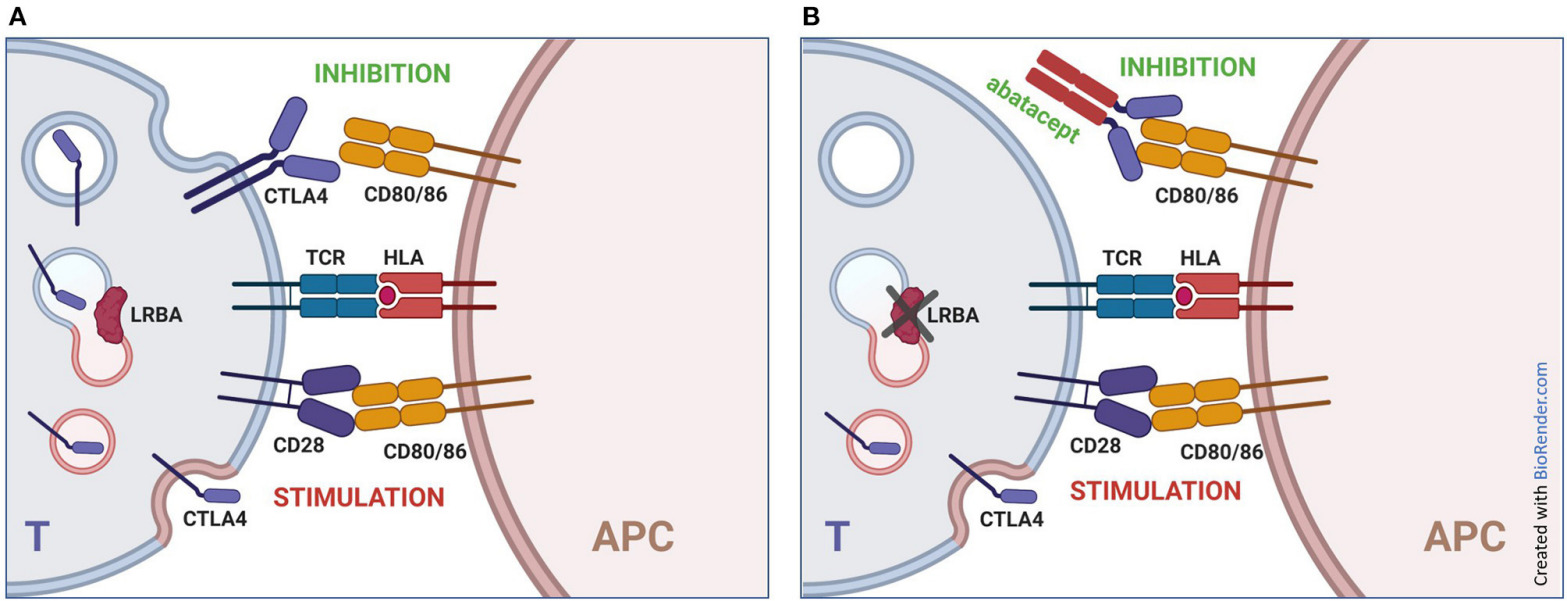
Mechanism of action of abatacept in LRBA deficiency. **(A)** normal immune responses are based on a balance between stimulatory signals (interaction of CD28 on T lymphocytes with CD80 and CD86 on antigen presenting cells) and inhibitory signals (block of CD80 and CD86 by CTLA4 expressed by lymphocytes). This balance is particularly critical in regulatory T cells, which can express higher levels of CTLA4. **(B)** biallelic mutations in LRBA result in impaired recycling of CTLA4 to the membrane, leading to reduced expression of the protein and increased lymphocyte stimulation. The addition of abatacept (CTLA4-Ig) partially compensates the lack of cell-bound CTLA4. T, T cell; APC, Antigen Presenting Cell.

Several immunomodulators have been proposed, focusing on the various clinical manifestations of the disease, including glucocorticoids, sirolimus, abatacept, mycophenolate mofetil, cyclosporin, azathioprine. Among these, abatacept can be considered a precision therapy, as it partially surrogates the lower CTLA4 expression (Figure 4). Accordingly, abatacept allowed a partial or complete control of disease in most patients, both reducing autoimmune phenomena and rescuing humoral immunity and in some cases was a good bridge therapy before HSCT (7, 32). In a large series treated with abatacept, autoimmune cytopenias improved in 5/6 patients, gastrointestinal inflammation in 7/10, lymphoproliferation in 10/14 (3). Lo et al. also described a normalization of immunoglobulins levels in three patients with hypogammaglobulinemia treated with abatacept (7). However, in some reports, the treatment with abatacept was not able to control adequately the disease (33).

In our case, sirolimus had been used with partial benefit on the ALPS-like lymphoproliferation, even if previous reports showed promising results (34). Only abatacept allowed a consistent and persistent improvement of the gastritis, allowing weaning from glucocorticoids and proton pump inhibitors.

With this report we would stress the importance of prompt investigations for genetic immune dysregulation disease in children with early onset gastroenterological autoimmune disorders, as recommended by several studies (20, 29, 35). The simultaneous presence of autoimmune manifestations and defective response to infections or lymphoproliferative features increase the suspicion index of an immune dysregulation disease (29, 31). However, even an isolate early onset atypical autoimmune disorder should be carefully considered to be underpinned by a monogenic defect. Next generation sequencing has made easier a prompt diagnosis, which in some cases can pave the way to meaningful therapies, from HSCT to abatacept in LRBA and CTLA4 deficiency.

## Data Availability Statement

The raw data supporting the conclusions of this article will be made available by the authors, without undue reservation.

## Ethics Statement

The studies involving human participants were reviewed and approved by Comitato Etico Indipendente Regione Friuli Venezia Giulia. Written informed consent to participate in this study was provided by the participants' legal guardian/next of kin.

## Author Contributions

VB wrote the manuscript. EV did immunological investigations and discussed the manuscript. MG performed genetic analysis and corrected the manuscript. AP performed transcriptomic studies and discussed the manuscript. LGD did expression studies. AT coordinated the study and revised the manuscript. SL critically revised the work and corrected the draft. MB coordinated gastroenterological studies and cared for the patient. All authors contributed to the article and approved the submitted version.

## Conflict of Interest

The authors declare that the research was conducted in the absence of any commercial or financial relationships that could be construed as a potential conflict of interest.
