# Supplementary material for: Case Report: Refractory Autoimmune Gastritis Responsive to Abatacept in LRBA Deficiency
Source: Front Immunol. 2021 Feb 26;12:619246. doi: 10.3389/fimmu.2021.619246 (PMC7952427; doi:10.3389/fimmu.2021.619246)

Supplementary Material

**Genetic analysis**

A custom-made panel of 10 genes (*CTLA4*, *IL21*, *IL21R*, *LRBA*, *NFNB2*, *PIK3CD*, *PIK3R1*, *RAG1*, *TNFRSF13B*, *TNFRSF13C*), responsible for common variable immunodeficiencies (CVID), was sequenced by Next Generation Sequencing (NGS) on PGM-Ion Torrent platform (Thermo Fisher Scientific). Torrent SuiteTM software v5.12 analyzed the signal processing and the free software wANNOVAR annotated the output file (http://wannovar.wglab.org/). All variants were further filtered based on type of mutation (non-synonymous, nonsense, frameshift and splicing about 10 nucleotides from the splice site), minor allele frequency (MAF) (absent if never described, <0.02 if recessive inheritance model or <0.001 if dominant inheritance model) (https://gnomad.broadinstitute.org/), damaging prediction (1) and clinical phenotype correlation (OMIM and Human Gene Mutation Database professional).

Two novel compound heterozygous mutations have been identified in the *LRBA* gene (chr 4; NM_006726 - NP_006717) in exon 42 (c.C6415T p.R2139X) and in exon 49 (c.C7315T p.R2439X), causing a premature STOP-codon in both alleles. Variants were validated by Sanger Sequencing also in parents, resulting in heterozygous condition (mother was carrier for p.R2139X and father for p.R2439X). According to American College of Medical Genetics (ACMG) guidelines, these two mutations were classified as “pathogenic” (2). Variants are located in the PH and BEACH domain respectively. Literature data described the deleterious effect of other nonsense mutations localized in these two domains, that are able to abolish the protein production probably through a “nonsense mediated decay” mechanism (3,4).

**Immunological investigations**

*Lymphocyte subpopulations*

Lymphocyte subpopulations were analyzed on peripheral whole blood by flow cytometry and were shown normal except for an increase of CD3+CD4-CD8-TCRαβ positive cells (DNTαβ cells) in line with LRBA deficiency diagnosis.

*CTLA4 Expression*

Peripheral blood mononuclear cells (PBMCs) of patient and healthy control, obtained from heparinized blood samples by means of density gradient centrifugation, were stimulated with PMA and ionomycin (Cell Stimulation Cocktail 500X, Invitrogen) for 2 hours at 37°C.

Cells were stained with CD4 APC (Milteny) and CTLA4 PE (BD) to analyze CTLA4 expression in CD4+ T cells and in lymphocytes by flow cytometry (Supplementary figure 1).

Sample data were acquired using MACSQuant Analyzer 10 (Milteny Biotec) and were analysed with FlowLogic software (version 7.2.1, Inivai Technologies).

*LRBA Expression*

Intracellular expression of LRBA was determined in PBMCs at steady conditions and upon stimulation with 10 ng/ml for 72 hours as follows: 250.000 cells were first collected, washed and stained for extracellular CD69-APC (BD; cat. 555533, dilution 1:20) and for fixable viability dye (FVD eFluor506, eBioscience; cat. 65-0866, dilution 1:500) for 15 minutes at 4°C. Next, the cells were fixed and permeabilized for 20 minutes using 200 µl of BD Cytofix/Cytoperm solution (BD; cat. 554715), and then washed twice at 1700rpm during 2 minutes with 200 µl of 1X Perm/Wash Buffer (BD; cat. 554723). Subsequently, cells were resuspended in 50 µl of 1X Perm/Wash Buffer and stained with a rabbit polyclonal anti-LRBA antibody (Sigma; cat. HPA023597; dilution 1:400) for 30 minutes at 4°C. A secondary antibody F(ab´)2 donkey anti-rabbit IgG-PE (BD; cat. 558416, dilution 1:25) was added and incubated at 4°C for 30 minutes. Cells were then washed and acquired on a FACS Canto II (BD). Data analysis and calculation of the geometric mean fluorescence intensity (MFI) were performed using FlowJo X software (TreeStar Inc; USA). Gating strategy was done as follows: cells were first gated for mononuclear cells (SSC-A vs. FSC-A) and singlets (FSC-H vs. FSC-A). Then, living mononuclear cells were gated based on the negative staining for the fixable viability dye and separated in cell subsets according to their extracellular marker. Cell activation was confirmed by measuring CD69 expression. Finally, LRBA expression was analyzed as a univariate histogram (Supplementary figure 2).

**Transcriptomic profile**

Transcriptomic analysis was performed on peripheral whole blood cells of the patient by RNA sequencing.

Differential gene expression pipeline was run in comparison to a group of 3 healthy male subjects.

The most representative genes were selected by fold change greater than 2-fold increase/decrease and adjusted p-value <0.05 (5). Among the differentially expressed genes, LRBA showed an about 4-fold decrease compared to controls consistent with the hypothesis of “nonsense mediated decay” mechanism attributable to STOP-codon mutations.

Moreover, pathway enrichment analysis (6) was carried out to investigate possible overexpressed biological processes without showing any statistically significant results.

1. Li Q, Wang K. InterVar: Clinical Interpretation of Genetic Variants by the 2015 ACMG-AMP Guidelines. *Am J Hum Genet*. (2017) 100:267-280. doi:10.1016/j.ajhg.2017.01.004.
2. Richards S, Aziz N, Bale S, Bick D, Das S, Gastier-Foster J,et al. ACMG Laboratory Quality Assurance Committee. Standards and guidelines for the interpretation of sequence variants: a joint consensus recommendation of the American College of Medical Genetics and Genomics and the Association for Molecular Pathology. *Genet Med*. (2015) 17:405–424. doi: 10.1038/gim.2015.30
3. de Valles-Ibanez G, Esteve-Sole A, Piquer M, Gonzalez-Navarro EA, Hernandez-Rodriguez J, Laayouni H, et al. Evaluating the Genetics of Common Variable Immunodeficiency: Monogenetic Model and Beyond. *Front Immunol*. (2018) 9:636. doi:10.3389/fimmu.2018.00636.
4. Charbonnier LM, Janssen E, Chou J, Ohsumi TK, Keles S, Hsu JT, et al. Regulatory T-cell deficiency and immune dysregulation, polyendocrinopathy, enteropathy, X-linked-like disorder caused by loss-of-function mutations in LRBA. *J Allergy Clin Immunol*. (2015) 135:217-227. doi:10.1016/j.jaci.2014.10.019.
5. Love MI, Huber W, Anders S. Moderated estimation of fold change and dispersion for RNA-seq data with DESeq2. *Genome Biol*. (2014) 15:550. doi:10.1186/s13059-014-0550-8.
6. Szklarczyk D, Gable AL, Lyon D, Junge A, Wyder S, Huerta-Cepas J, et al. STRING v11: protein-protein association networks with increased coverage, supporting functional discovery in genome-wide experimental datasets. *Nucleic Acids Res*. (2019) 47:D607-D13. doi:10.1093/nar/gky1131.

## Supplementary Figures

**
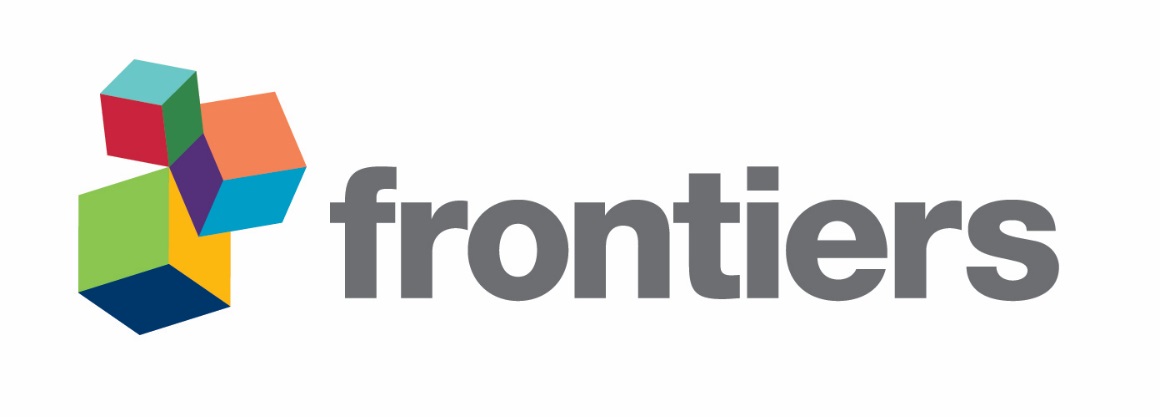
**

**Supplementary figure 1.** CTLA4 expression in resting and PMA-ionomycin activated PBMC. Manual gating strategy was performed in order to analyze CTLA4 expression in CD4+ T cells (A) and in lymphocytes (B). Green and yellow: resting and stimulated cells from healthy control; Blue and Red: resting and stimulated cells from patient.

**
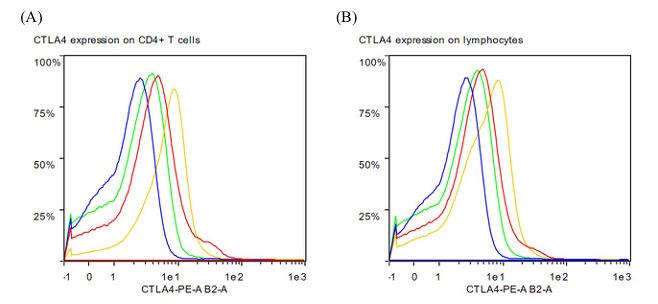
**

**Supplementary figure 2.** LRBA expression in PHA activated PBMC. Manual gating strategy was performed in order to analyze LRBA expression in CD69+ cells of travel healthy control (A), internal laboratory control (B) and patient (C). Fill Red (D): stimulated cells from patient; Blue line (D): stimulated cells from travel healthy control; Black line (D): stimulated cells from internal laboratory control.


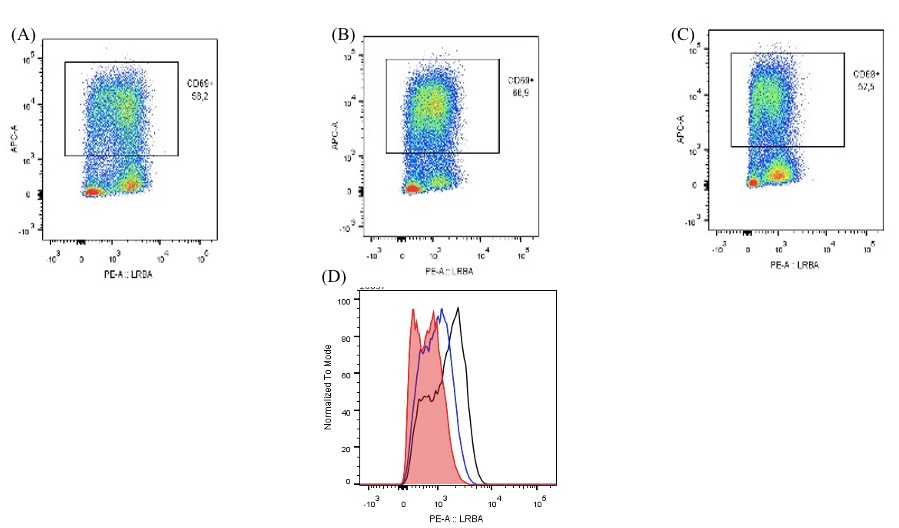

Supplement: Supplementary file 1 [file Data_Sheet_1.docx]
